# Supplementary material for: Tgfβ signaling is required for tenocyte recruitment and functional neonatal tendon regeneration
Source: eLife. 2020 Jun 5;9:e51779. doi: 10.7554/eLife.51779 (PMC7324157; doi:10.7554/eLife.51779)
Supplement: Supplementary file 1. [file elife-51779-supp1.docx]

**Supplemental Table 1: Primer sequences for real time qPCR**

| **Gene** | **FWD Primer** | **REV Primer** |
| --- | --- | --- |
| *Tgfbr2* | CCAAGTCGGATGTGGAAATGG | TGTCGCAAGTGGACAGTCTC |
| *Tgfb1* | ACGTGGAAATCAACGGGATCA | AGAAGTTGGCATGGTAGCC |
| *Tgfb2* | CCCTCCGAAAATGCCATCC | TGCTATCGATGTAGCGCTGG |
| *Tgfb3* | ATGACCCACGTCCCCTATCA | CAGACGGCCAGTTCATTGTG |
